# Identification and Characterization of *CDH1* Germline Variants in Sporadic Gastric Cancer Patients and in Individuals at Risk of Gastric Cancer

**DOI:** 10.1371/journal.pone.0077035

**Published:** 2013-10-29

**Authors:** Marica Garziera, Vincenzo Canzonieri, Renato Cannizzaro, Silvano Geremia, Laura Caggiari, Mariangela De Zorzi, Stefania Maiero, Enrico Orzes, Tiziana Perin, Stefania Zanussi, Paolo De Paoli, Valli De Re

**Affiliations:** 1 Departement of Translational Research, Centro di Riferimento Oncologico (CRO), National Cancer Institute, Aviano, Pordenone, Italy; 2 Pathology Unit, Centro di Riferimento Oncologico (CRO), National Cancer Institute, Aviano, Pordenone, Italy; 3 Gastroenterology Unit, Centro di Riferimento Oncologico (CRO), National Cancer Institute, Aviano, Pordenone, Italy; 4 CEB-Centre of Excellence in Biocrystallography, Department of Chemical Sciences, University of Trieste, Trieste, Italy; 5 Microbiology-Immunology and Virology Unit, Centro di Riferimento Oncologico (CRO), National Cancer Institute, Aviano, Pordenone, Italy; 6 Scientific Director, Centro di Riferimento Oncologico (CRO), National Cancer Institute, Aviano, Pordenone, Italy; Ohio State University Medical Center, United States of America

## Abstract

**Objective:**

To screen and characterize germline variants for E-cadherin *(CDH1)* in non-hereditary gastric cancer (GC) patients and in subjects at risk of GC.

**Methods:**

59 GCs, 59 first degree relatives (FDRs) of GC, 20 autoimmune metaplastic atrophic gastritis (AMAGs) and 52 blood donors (BDs) were analyzed for *CDH1* by direct sequencing, structural modelling and bioinformatics. Functional impact on splicing was assessed for intronic mutations. E-cadherin/β-catenin immunohistochemical staining and E-cadherin mRNA quantification using RT-PCR were performed.

**Results:**

In GCs, 4 missense variants (p.G274S; p.A298T; p.T470I; p.A592T), 1 mutation in the 5′UTR (−71C>G) and 1 mutation in the intronic IVS12 (c.1937-13T>C) region were found. First pathogenic effect of p.A298T mutation was predicted by protein 3D modelling. The novel p.G274S mutation showed a no clear functional significance. Moreover, first, intronic IVS12 (c.1937-13T>C) mutation was demonstrated to lead to an aberrant *CDH1* transcript with exon 11 deletion. This mutation was found in 2 GCs and in 1 BD. In FDRs, we identified 4 variants: the polymorphic (p.A592T) and 3 mutations in untranslated regions with unidentified functional role except for the 5′UTR (−54G>C) that had been found to decrease *CDH1* transcription. In AMAGs, we detected 2 alterations: 1 missense (p.A592T) and 1 novel variant (IVS1 (c.48+7C>T)) without effect on *CDH1* splicing. Several silent and polymorphic substitutions were found in all the groups studied.

**Conclusions:**

Overall our study improves upon the current characterization of *CDH1* mutations and their functional role in GC and in individuals at risk of GC. Mutations found in untranslated regions and data on splicing effects deserve a particular attention like associated with a reduced E-cadherin amount. The utility of *CDH1* screening, in addition to the identification of other risk factors, could be useful for the early detection of GC in subjects at risk (i.e. FDRs and AMAGs), and warrants further study.

## Introduction

Gastric cancer (GC) remains the fourth most common malignancy worldwide, even though its incidence and associated mortality rates have decreased in recent decades. GC prognosis is closely related to the stage of disease at diagnosis [1]. Early onset gastric cancer (EOGC) is defined as GC presenting at the age of 45 or younger [2] and has a poor overall survival [3], [4]. Most GCs are sporadic and often develop following *Helicobacter pylori* (HP)-associated gastritis [5], [6]. However, familial aggregation studies also stress the importance of a genetic predisposition in the sporadic development of GC. Frequency of familial gastric aggregation is about 10%.

The most widely accepted GC histopathological classification (Lauren's classification) [7] distinguishes two types of GC: intestinal type and diffuse type. Diffuse GC shows a greater hereditary basis and a generally worse prognosis as compared with the intestinal subtype [8].

*CDH1* gene coding for the E-cadherin has been identified to have a causative role in about 30%–50% of hereditary diffuse GC (HDGC), an autosomal dominant GC and lobular breast cancer susceptibility syndrome constituting 1–3% of familial clustering of GCs [9], [10] and in diffuse GC subtype [11]. *CDH1* germline mutations (such a mutation is passed on every cell in the offspring's body) are specifically associated with HDGC (about 30%–40% of cases); large *CDH1* deletions have been found in about 6.5% of cases [12]. Familial intestinal gastric cancer (FIGC) with a positive family history have also been described but so far, no germline *CDH1* defects have been associated with FIGC or intestinal GCs. This lack of evidence of *CDH1* mutations in the intestinal subtype has led to the hypothesis that familial clustering in these cases is determined by shared environmental factors, as opposed to an inherited genetic predisposition. However, recent data demonstrate that *CDH1* somatic alterations (such alterations accumulate in the cancer cells of the body over a person's lifespan) are as frequent in intestinal as in diffuse GC [13], suggesting an important role of *CDH1* in both the histotypes. Nonetheless, the exact prevalence of *CDH1* germline alterations in intestinal GCs is still unknown. *CDH1* promoter hypermethylation is the most common second genetic hit in the GC carcinogenic process [14], [15]. *CDH1* mutations are also associated with an increased susceptibility to invasive and metastatic [16], [17] colon, bladder, prostatic, breast and gynaecological cancers [18]–[20]. E-cadherin is a transmembrane glycoprotein that plays a role in maintaining epithelial tissue architecture by involving Ca^2+^ dependent cell-cell interactions [21], [22]. E-cadherin comprises a cytoplasmic domain, a short transmembrane domain and five extracellular repeat cadherin-like domains (EC1-5) that span exons 4–13 and contain highly conserved calcium-binding regions [23], [24] and conserved cysteines likely to form disulfide bridges [25].

In this study, we analysed *CDH1* germline mutations in a series of consecutive random GC cases and individuals at risk of GC; mainly first degree GC-Relatives (FDRs) and autoimmune metaplastic atrophic gastritis (AMAG) patients directed to our institute for gastrointestinal symptoms and endoscopic evaluation. To explore the role of E-cadherin expression, structural, functional and immunohistochemical analyses were performed in samples with a *CDH1* germline mutation. The aim of the present study was to evaluate the prevalence and characterize *CDH1* germline mutations in a series of consecutive sporadic GC patients lacking the criteria of HDGC classification, and in a selected population at risk of GC development, to test its utility as a marker to improve early tumor detection. Data obtained could be used to develop a tool that rapidly and cheaply detects *CDH1* mutations mainly present in our population.

## Results

### Patient characterization and *CDH1* germline genetic screening

Clinical and histopathological features of GC, FDR and AMAG subjects are summarized in Tables 1 and 2. Among the 59 GC patients, 2 (3.4%) have a family history of GC (S15 is the brother of S16) without meeting the criteria for hereditary diffuse GC, as defined by the International Gastric Cancer Linkage Consortium (IGCLC) at the time of sample collection. In our GC series, 5 sporadic early GC patients (≤45 years old) were present, but no *CDH1* alterations were found in these patients. The median age of the FDRs was 49 years (range, 28–78 years) and for AMAGs 56 years (range, 31–72 years). Among the 59 GC patients, 16 subjects had a first degree relative included in the study (16/59 FDRs). FDRs and AMAGs came to our institution for a gastroenterology visit and gastroscopy exam, they manifested various symptoms, but neither cancer nor intestinal metaplasia/dysplasia was present in these subjects.

**Table 1:** Clinicopathological characteristics of patients with gastric cancer.

Variable	
**Tumor classification (Lauren)**	
*intestinal type*	19
*diffuse type*	26
*mixed type*	12
*indeterminate type*	2
**Location**	
*Proximal*	17
*Distal*	39
*Linitis plastica*	3
**Stage**	
*Not resected*	21
*0*	3
*1*	8
*2*	12
*3*	6
*4*	2
*Not classified*	2
**Operation (Type of resection)**	
*Not resected*	21
*T1*	8
*T2*	11
*T3*	15
*T4*	2
*Not classified*	2
**Lymph node status**	
*Not resected*	21
*N0*	15
*N1*	10
*N2*	3
*N3*	8
*Not classified*	2

**Table 2:** Patients characteristics and clinical presentation.

	GCs, n = 59	FDRs, n = 59	AMAGs, n = 20
**Age-years (± SEM)**	60.7±1.65	45.7±1.68	56.7±2.44
*Range*	19–85	23–78	31–70
*Gender*			
*male*	34	29	3
*female*	25	30	17
***H. Pylori*** ** Infection**			
*Positive*	20	25	2
*Negative*	34	29	16
*Nd*	5	4	2
**Gastropanel**			
*PGI (±SD)*	152.8 (±139.3)	94.0 (±41.1)	29.0 (±75.9)
*PG2 (±SD)*	23.2 (±20.5)	11.2 (±7.2)	10.9 (±4.5)
*G-17 (±SD)*	21.6 (±22.1)	13.1 (±19.5)	301.0 (±231.5)

**Abbreviations:** GCs, patients with Gastric Cancer; FDRs, First Degree Relatives without neoplasia; AMAGs, Autoimmune Metaplastic Atrophic Gastritis patients without neoplasia; SEM, Standard Error of the Mean; H, Helicobacter; PGI, Pepsinogen I; PG2, Pepsinogen II; G-17, Gastrin-17; Nd, Not determined; SD, Standard Deviation.

*CDH1* genetic screening results are listed in Table 3 with *new* mutations: 1 intronic (ID 5), 1 missense (ID 10), and 2 silent (ID 13 and ID18). Overall we found 4 variants, which code for an amino acid (AA) substitution (1 novel (ID10) and 3 previously reported in other populations (ID 11, ID 12, ID 15), 1 in the 5′near gene region and 2 mutations in the untranslated (UTR) regulatory element (ID 1, ID 3, already reported) and 6 substitutions in intronic regions (three mutations: ID 5, ID 9, ID 17; three intronic polymorphic variants: ID 4, ID 6, ID 8, probably with no effect on GC cancerogenesis). No deletions or insertions were found in the exon boundaries.

**Table 3:** Analysis of *CDH1* germline mutations found in 190 individuals.

ID	GenReg	(c)	(p)	GCs# (Histotype)	FDRs#	AMAGs#	BDs#	Freq ##	Subject code	rs	Ref
D 1	5′UTR	−176C>T	UTR	0	1	0	0	0.90%	S112	34500817°°	[65]
ID 2	5′UTR	−71C>G	UTR	3(I;M;D)	0	0	0	0.0%	S25;S27;S30	34033771°°	[45]
ID 3	5′UTR	−54G>C	UTR	0	1	0	0	0.90%	S63	5030874°°	[54]
ID 4	IVS1	48+6C>T	Intr	9(5I;1M;2D;1Ind)	12	6	8	14.15%	Polym.variant	3743674°°	[49]
ID 5	IVS1	48+7C>T	Intr	0	0	1	0	0.0%*	S121	*New* mut	
ID 6	IVS1	48+62_63d/i	Intr	48(24D;12I;11M;1Ind)	45	14	43	77.45%	Polym.variant	74406246°°°	[66]
ID 7	Ex 3	345G>A	T115T	3(D;M;M)	1	1	1	0.09%	S6;S15;S47;S70;S120;S183	1801023°°	[47]
ID 8	IVS4	531+10G>C	Intr	0	4	0	1	4.50%	S74;S75;S76;S107;S158	33963999°°	[47], [48]
ID 9	IVS4	532-18C>T	Intr	0	1	0	0	0.90%	S97	200673941°°	[35], [56]
ID 10	Ex 6	820G>A	G274S	1(M)	0	0	0	0.0%*	S38	*New* mut	[26] **
ID 11	Ex 7	892G>A	A298T	1(M)	0	0	0	0.0%	S47	142822590°°	[27]
ID 12	Ex 10	1409C>T	T470I	1(D)	0	0	0	0.0%	S39	no rs	[28]
ID 13	Ex 10	1416C>T	T472T	1(D)	0	0	0	0.0%*	S4	*New* mut	
ID 14	Ex 11	1680G>C	T560T	3(D;D;I)	1	0	0	0.90%	S9;S11;S50	35741240°°	[47]
ID 15	Ex 12	1774G>A	A592T	1(M)	1	1	1	1.80%	S49;S115;S125;S156	35187787°°	[35], [41]
ID 16	Ex 12	1896C>T	H632H	0	2	1	2	3.60%	S65;S118;S126;S187;S188	33969373°°	[34], [48]
ID 17	IVS12	1937-13T>C	Intr	2(D;D)	0	0	1	0.90%	S10;S46;S190	2276330°°	[46]–[48]
ID 18	Ex 13	2073C>T	A691A	1(I)	0	0	0	0.0%*	S48	*New* mut	
ID 19	Ex 13	2076T>C	A692A	24(12D;7I;4M;1Ind)	29	10	23	44.34%	Polym.variant	51801552°°	[46]
ID 20	Ex 14	2253C>T	N751N	1(M)	4	1	3	6.31%	S38;S83;S86;S91;S92;S123;S167;S186;S190	33964119°°	[46], [48]
ID 21	Ex 14	2292C>T	D764D	0	0	0	1	0.90%	S161	61747636°°	[48]
ID 22	Ex 16	2634C>T	G878G	0	0	0	1	0.90%	S188	2229044°	[47], [48]

**Legend:** ID = Variant code; GenReg = Gene Region; (c) = nucleotide code position; (p) = protein code position; BDs, Blood Donors (n = 52); Freq = Frequency; rs = refSNPcluster; Ref = Reference; Ex = Exon; UTR = Untranslated; d/i = deletion/insertion; Intr = Intronic; I = Intestinal, D = Diffuse, M = Mixed, Ind = Indeterminate, histotypes; Polym. = Polymorphic; mut = mutation. Other abbreviations are described in Table 2 Abbreviations list.

#, in the column are indicated the number of subjectss having the ID mutation.

##, Frequency of variants was calculated in 111 healthy subjects (52BDs+59FDRs). 16 FDRs were first degree relatives of our GC series, when one of the variant was present in GC and in its related FDR case, we excluded the FDR individual from the frequency calculation.

* : new mutation frequency was evaluated by extending BD subjects analysis from 52 to 108 individuals.

** : novel mutation with uncertain pathogenetic effect that we have previously reported [26].

° : variant with frequencies in NHLBI exome sequencing project (ESP).

°° : variant with frequencies in NHLBI exome sequencing project (ESP) and 1000 Genomes project.

°°° : variant with frequencies in 1000 Genomes project.

Other alterations resulted common polymorphisms (frequency of at least 1% in the population) or silent mutations that code for the same amino acid than the original strand. None statistical association was observed among the four groups of patients tested for ID 4, ID 6 or ID 19 variants.

RefSNP (rs) numbers to identify genetic variants previously published as well as their reported frequencies (NIEHS Environmental Genome Project, Seattle, WA (**URL:** http://evs.gs.washington.edu/niehsExome/ Accessed August 2013); ftp://ftp.1000genomes.ebi.ac.uk/vol1/ftp/phase1/analysis_results/paper/ **Accessed** December 2012) are reported in Table 3.

All *CDH1* variants were in heterozygous state except for the ID 4 and ID 19 in which a homozygous state was also detected.

**Figure 1:**
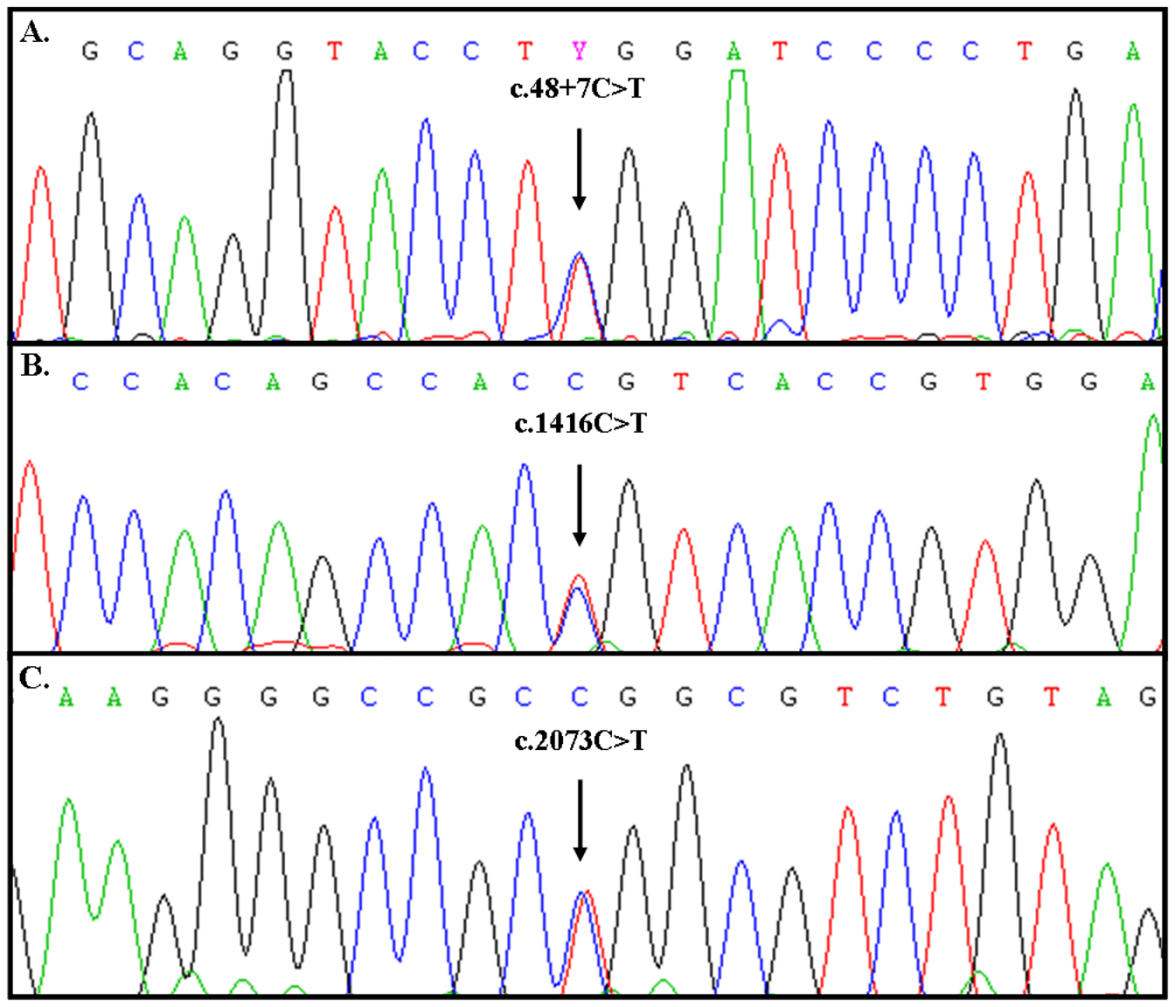
Sequencing chromatograms showing the novel *CDH1* germline mutations. (A) ID 5, new intronic mutation close to exon 1 (IVS1 c.48+7C>T) found in one patient affected by AMAG (S121). (B) ID 13, silent mutation (c.1416C>T) with conserved Ala residue at position 472 in *CDH1* exon 10 (p.T472T) found in one GC (S4). (C) ID 18, the silent mutation (c.2073C>T) with conserved Ala residue at position 691 in *CDH1* exon 13 (p.A691A) found in one GC (S48).

Frequency of mutations and variants were calculated in subjects without GC or AMAG disease (52BDs+59FDRs, n = 111). Sixteen FDRs were first degree relatives of our GC series; when one of the variant was present in GC and its related FDR case, we excluded the FDR individual from the frequency calculation. ID4 for example, was present in 5 FDRs related to our GC of our series; therefore the control population frequency changed from total 111 to 106 individuals (7FDRs+8BDs/106; 14.5%). Figure 1 illustrates sequencing chromatograms of the novel mutations we have found. We have previously reported the ID 10 chromatogram in another paper [26].

### Bioinformatic predictive role and structural modelling results of missense variants found

**Figure 2:**
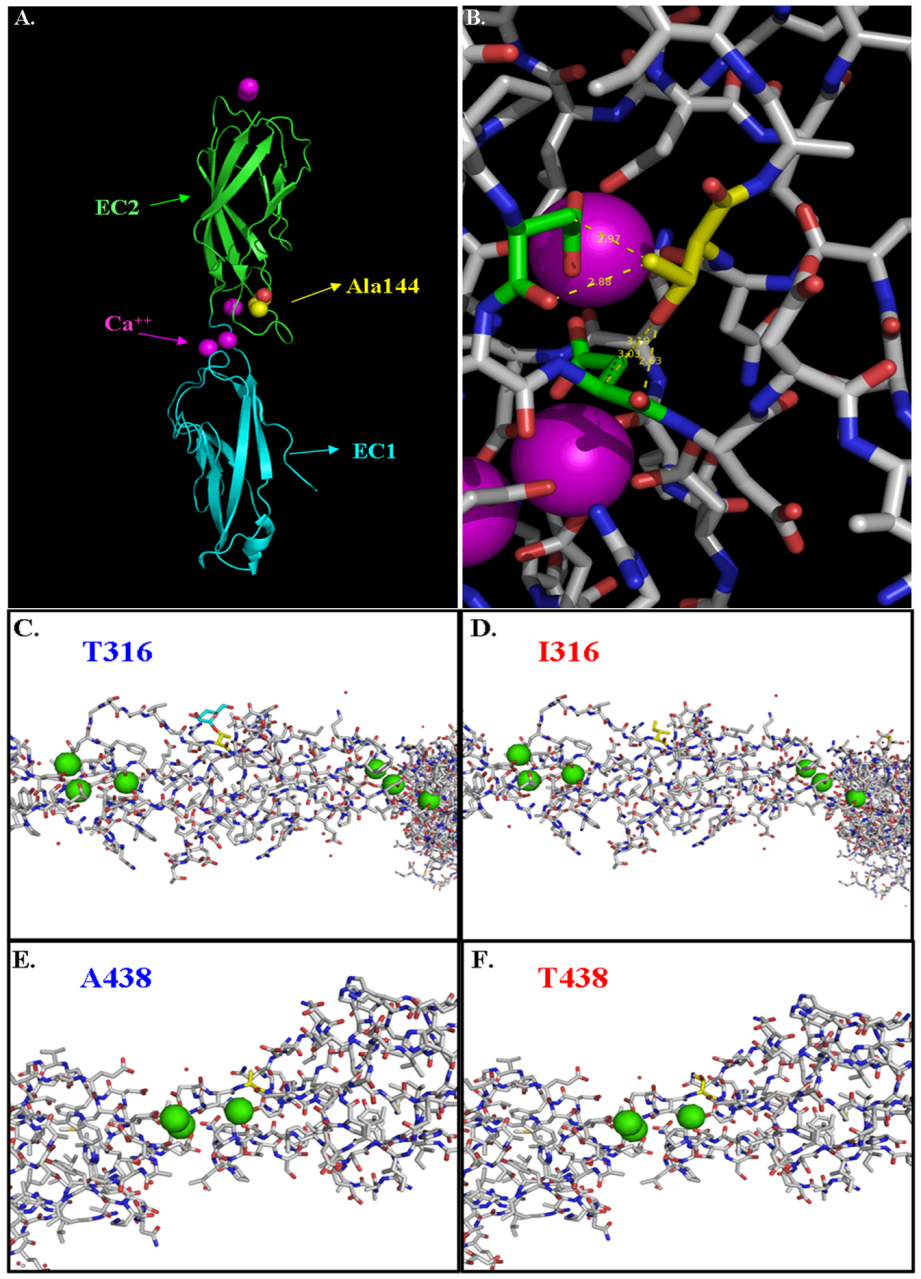
Structural modelling of the extracellular domains of the E-cadherin protein. In A–B PyMOL and Coot softwares representations of EC1–EC2 protomers based on human sequence (PDB code: 2O72); in C–F, EC3 and EC4 protomers based on murine E-cadherin (PDB code: 3Q2W) by Coot software. (A) Cartoon representation highlights A144 (*yellow*) position: A144 is near to calcium sites (*purple*) and in proximity of the dimerization interface between EC1 (*blue*)-EC2 (*green*) domains. (B) Structural representation of A144T substitution in EC2 domain. Position of AT144 is spotlighted in yellow. Threonine in position 144 is quite dramatic for local structure because it interacts with two Aspartic acid residues (*green*) directly involved in calcium sites, and these bond lengths are particularly stressed being less than 3 Å. (C) The T316 (*yellow*) is *O*-glycosilated (*blue*) and present on the surface of EC3 domain. (D) The hydrophobic lateral chain of I316 (*yellow*) cannot be *O*-glycosilated and promotes protein-protein interactions. (E) The methylenic side chain of A438 (*yellow*) allows conformational freedom on the surface of EC4 domain. (F) T438 (*yellow*) substitution is not dramatic for local structure but it could represent a potential site for post-translational modifications. Calcium ions are highlighted (*green*).

The missense mutated residues we found are all localized to the E-cadherin extracellular domain. The codon position in the immature and mature (after the N-terminal cleavage) proteins and data from the PolyPhen-2 and SIFT *in silico* analyses are reported in Table 4. All four missense variants are potentially damaging by PolyPhen-2, but only the p.A298T (ID 11) and p.A592T (ID 15) substitutions may affect protein function by SIFT analysis (Table 4). The p.G274S (ID 10) that we recently described [26], however, does not perturb the local environment, but introduces a potential residue for phosphorylation and glycosylation that may have possible effects on the stability and integrity of E-cadherin as we hypothesized [26]. The pathogenetic effect of ID 11 substitution was previously established [27], but was here first demonstrated by structural analysis. As illustrated in Figure 2A, the AA change in exon 7 of the p.A298T (ID 11) is positioned near the interactive region between protomers EC1 and EC2. Thus, the alanine-threonine polar residue substitution may drive H-bond formation through its oxydrilic group and this may interfere with the local structure of the protein in a region that is fundamental for Ca^2+^ interactions. Threonine in position 144 is sterically demonstrated obtrusive because it interacts with two aspartic acid residues (Asp136 and Asp 138) that are directly involved in Ca^2+^ binding. Moreover, the bond lengths are particularly stressed, being less than 3 Å (Figure 2B).

**Table 4:** Summary of germline missense variants detected in *CDH1* gene and predictedfunctional effect by SIFT and PolyPhen tools.

*CDH1* Ex	(c)	(p) pre	(p) mature	EC	PolyPhen Analysis	SIFT BlinkAnalysis (score)	Ref	Subject code
6	820G>A	G274S	G120S	EC2	Probably damaging	Tolerated (0.57)	26	S38
7	892 G>A	A298T	A144T	EC2	Probably damaging	Affect Protein Function (0.02)	27	S47
10	1409 C>T	T470I	T316I	EC3	Probably damaging	Tolerated (0.16)	28	S39
12	1774 G>A	A592T	A438T	EC4	Possibly damaging	Affect Protein Function (0.04)	40–43	S49;S115;S125;S156

**Legend:** (p) pre = protein code position in pre-protein; (p) mature = protein code positionin mature protein; EC = Extracellular Cadherin like domain. Other abbreviations are listed in Table 3 legend.

As regard to the remaining two missense mutations, they have a less clear functional effect as also reported in Table 4. p.T470I (ID 12) substitution [28] changes the AA surface of extradomain EC3 in the mature protein (Figure 2C). In both the murine E-cadherin and N-cadherin sequence (PDB code: 3Q2W) threonine is usually found *O*-glycosylated suggesting an important role for this residue in the structure of the protein. However, as showed in the Figure 2D, the change to isoleucine, a non-polar AA with a hydrophobic side chain that cannot undergo post-translational modification, suggests no particular intermolecular tension. We hypothesize that in the extracellular medium, the presence of an isoleucine residue at the same position than threonine may favour protein-protein interactions, and this mutation could thus assume a protective significance. The last mutation reported, p.A592T (ID 15), was found in all groups tested (see Table 3), suggesting an improbable effect on GC pathogenesis. In this case, Alanine on the extradomain EC4 of the mature E-cadherin (Figure 2E) provides conformational freedom, even when in proximity of the Ca^2+^ binding sites. A threonine substitution here has a limited effect on the local structure and torsional angles of the protein. However, we can not exclude that the oxydrilic lateral chain could be post-translational modified in particular situation and thus influence the structure and function of the *CDH1* (Figure 2F).

### Transcript analysis of intronic germline mutations

To explore if intronic mutations detected in our GC series (Table 3) could potentially induce an effect on splicing, we performed *CDH1* transcription analysis. Polymorphic and silent variants were excluded from this analysis since they probably have no pathogenic role. cDNA produced from peripheral blood of the selected GC individuals harbouring intronic ID 5, ID 9 or ID 17 mutations (Table 3) were compared to that from two healthy blood donors, one only having the same ID 17 mutation as GC patients (BD code S190), and another (BD code S189) without *CDH1* mutation.

**Figure 3:**
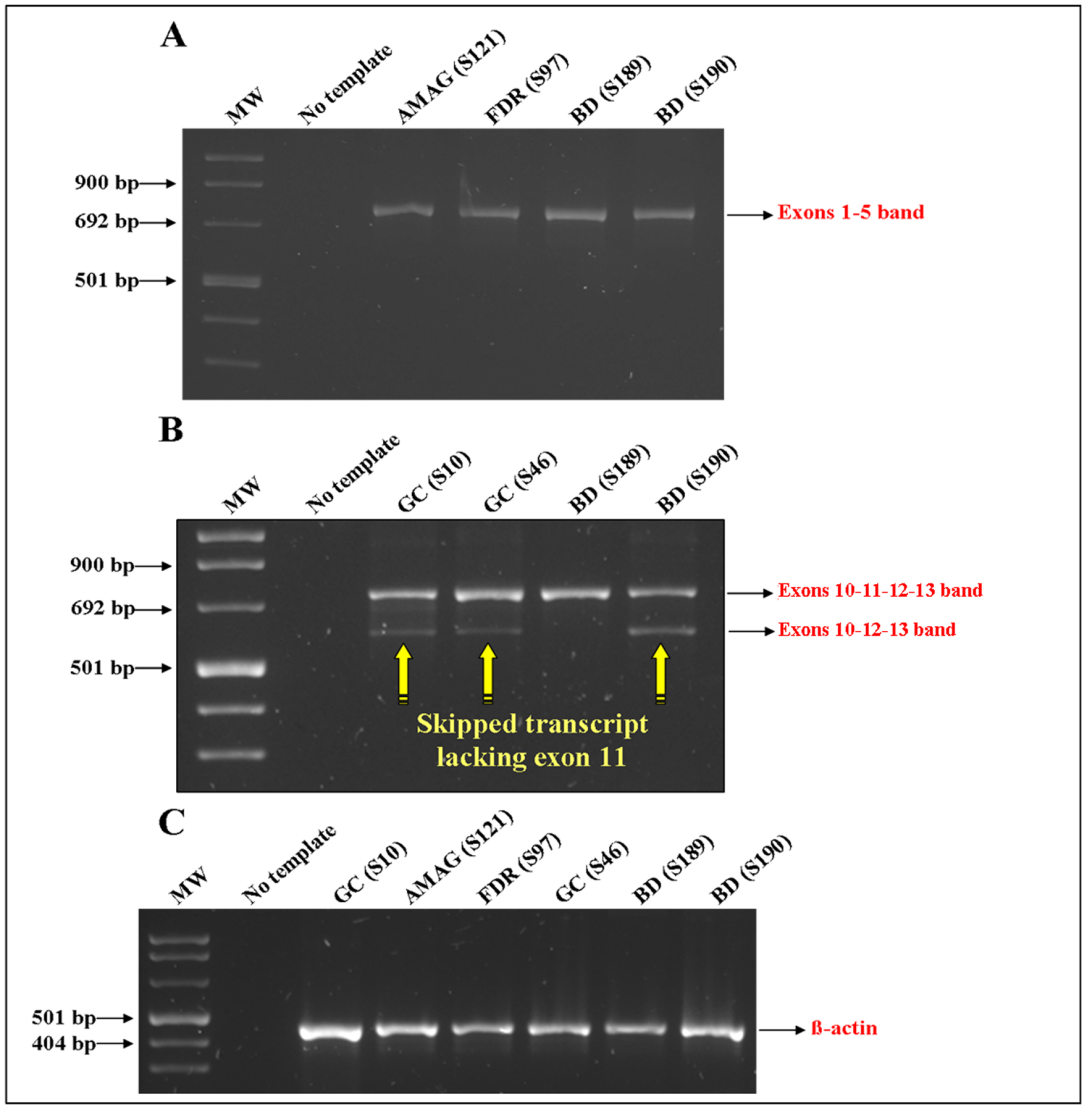
Evaluation of potential effects on splicing on *CDH1* gene of the intronic variants found. Total mRNA was extracted from PBMCs of each represented sample and retrotrascribed to single strand cDNA to amplify: (A) Exons 1–5 of about 768 bp. In the second lane AMAG (S121) patient carrying the ID 5 mutation (IVS1 c.48+7C>T); in the third lane FDR (S97) carrying the ID 9 mutation (IVS4 c.532-18C>T); (B) Exons 10–13 of about 686 bp. GC (S10), GC (S46) and BD (S190) carrying the ID 17 mutation (IVS12 c.1937-13T>C). Yellow arrows evidence a smaller band corresponding to aberrant transcripts lacking the exon 11; (C) β-actin was used as internal amplification control. MW: 1 Kb DNA ladder. PCR products were run in a 4% agarose and gel stained with SYBR Green dyef

For the ID 5 and ID 7 intronic mutations, we amplified the region covering part of exon 1 to part of exon 5, for ID 17 mutation, exon 10 to 13 (Figure 3). The RT-PCR exon 1 to 5 fragments showed no differences when run on 4% agarose gel (Figure 3A) nor after bidirectional sequencing (data not showed); by converse ID 17 intronic variant could affect splicing leading to an abnormal smaller *CDH1* transcript (Figure 3B). Upon isolation and sequencing, we found that the smaller band resulted in a skipped transcript lacking exon 11, with exon 10 directly joined to exon 12. This aberrant transcript was also detected in the BD S190 carrying the same germline substitution (Figure 3B).

### Analysis of *CDH1* protein abundance and mRNA expression level in subjects showing *CDH1* intronic mutations

**Figure 4:**
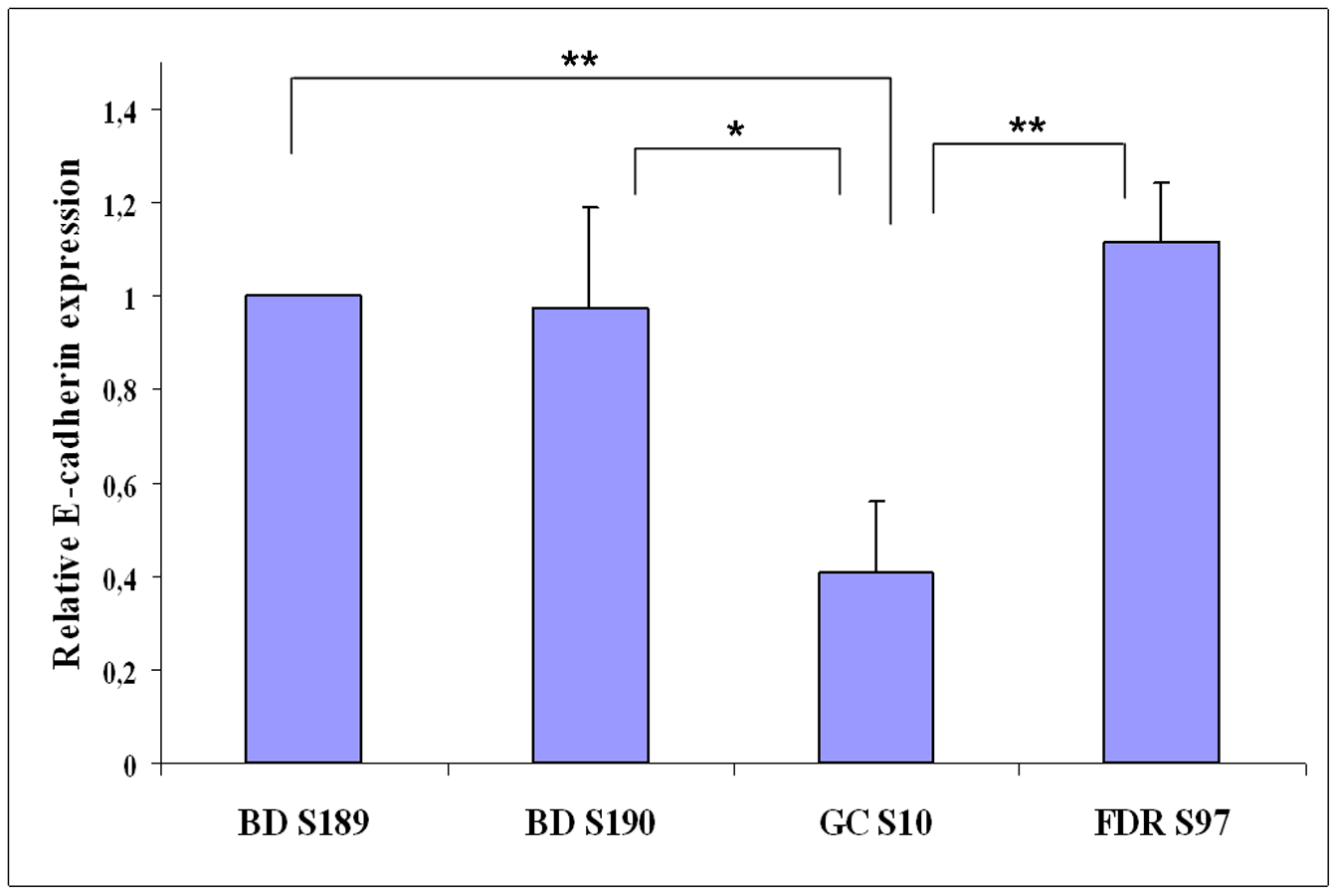
Graphic presentation of relative E-cadherin quantification by quantitative real time RT-PCR. Relative quantification of E-cadherin mRNA levels in EBV immortalized lymphocytes (LCL). LCLs were generated from the seeding of 2,5×10^6^ PBMCs immortalized by B.95.8 EBV and cultured in suspension. About 8 million of cells were harvested for each sample after immortalization. Patients tested were: GC S10 harbouring the ID 17 variant (IVS12 c.1937-13T>C), FDR S97 carrying the ID 9 (IVS4 c.532-18C>T), BD S190 with ID 17 and BD S189 without any *CDH1* mutation. S189 was considered as the reference (value of 1) Results are representative of three independent experiments. E-cadherin expression level was normalized normalized to β-actin Data are represented as means ± SD. *, p<0.05, **, p<0.01.

A comparison of E-cadherin mRNA expression level was performed from EBV immortalized lymphocytes obtained from the peripheral blood of S10 and S190 (mutation ID 17), S97 (ID 9) and S189 (no *CDH1* mutations) subjects. Subject S10 was affected by a gastric cancer, subject S97 is first degree relative of a patient with a gastric cancer (FDR), while S189 and S190 were both blood donors. We observed between the control blood donor (S189) having no *CDH1* mutation and patients, a relative strong decrease in E-cadherin expression (about 60%, Figure 4) in patient S10 having both ID 17 mutation and a GC, while only about a 2% reduction in the blood donor S190 having the same ID 17 mutation (*p*<0.05, with respect to GC S10). For S97 (mutation ID 9, FDR subject), we observed a similar E-cadherin expression as that in the control S189.

**Figure 5:**
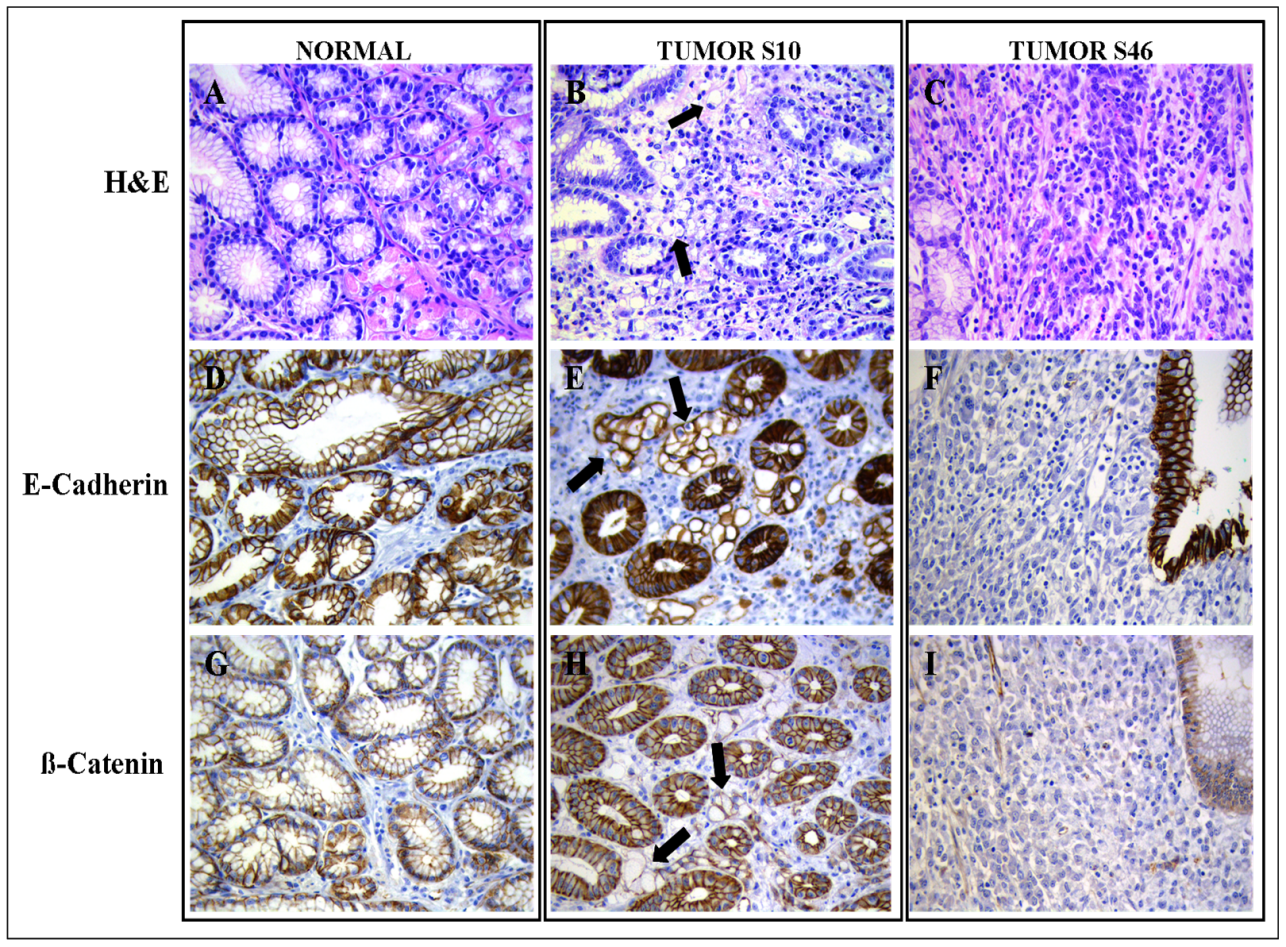
Hematoxylin and eosin stain gastric sections and immunohistochemical staining for E-cadherin and β-catenin. (A) Hematoxylin and eosin staining in normal gastric tissue. (B) Hematoxylin and eosin staining in the tumor tissue of GC S10 with signet ring cell carcinoma: signet ring cells are highlighted by black arrows. (C) Hematoxylin and eosin staining evidence the diffuse histotype of GC S46. (D) E-cadherin staining in normal gastric tissue. (E) Reduction of E-cadherin staining in the signet ring cells of GC S10 respect to adjacent normal cells; signet ring cells are highlighted by black arrows. (F) Loss of E-cadherin expression in the diffuse tumor cells of GC S46 compared to normal tissue (on the right side of the photomicrograph). (G) β-catenin staining in normal gastric tissue. (H) Weakly β-catenin staining in signet ring cells (black arrows) of GC S10. (I) Loss of β-catenin staining in the tumor tissue of GC S46 compared to normal tissue (on the right side of the photomicrograph). All the photomicrographs were taken at 400× magnification.

Immunohistochemical analysis on the tumor gastric tissue of intronic ID 17 case (patient code S10, Figure 5E) showed a reduced expression of membrane-bound E-cadherin in the signet ring tumor cells (black arrows), while both membrane and cytoplasmic staining were present in the normal epithelium. The same patient showed reduced β-catenin staining in the signet ring cells as compared with the strong expression of this protein in the normal adjacent cells (Figure 5H). The loss of both E-cadherin and β-catenin staining was also noticeable for the second patients (S46) having the same intronic ID 17 mutation and affected by GC too (Figure 5F and 5I, respectively for E-cadherin and β-catenin).

## Discussion

GC patients typically have a poor prognosis [29]. Identification of patients with an increased risk of developing GC and the early detection of GC are promising approaches to reduce the morbidity and mortality of GC. FDR of GC patients are known to have a 2–3 fold increased risk of GC, probably owing to exposure to the same environmental risk factors and/or to inherited susceptibility to cancer [30].

The parietal cells destruction found in AMAG combined with the important role of E-cadherin in epithelial polarity and gastric glandular architecture, suggests that germline alterations of *CDH1* could be an additional risk factor for GC development in AMAG patient [31].

In 1998, Guilford and colleagues described for the first time germline mutations of the *CDH1* gene [28]. Subsequently, different types of mutations have been reported in families of varying ethnicities with diffuse GC [32], [33]. The first *CDH1* germline mutation was described in an Italian family in 2006, in a patient who met the IGCLC criteria for HDGC [34]. However, very few studies report *CDH1* germline mutations in sporadic GC cases without familial aggregation or in subjects at risk of developing GC [35], [36]. Moreover, in these studies the functional effects of *CDH1* variants often are not investigated.

The strength of our study is the collection of 59 Caucasian patients with sporadic GC, 59 FDRs and 20 AMAGs who attended our gastroenterology service in the last years for gastric symptoms and a diagnosis or exclusion of a GC after endoscopic and histological tissue evaluation.

As summarized in Table 3, various different germline *CDH1* variants have been detected. In the 59 GC series, excluding the polymorphic and silent changes that probably have no pathogenic role, we found 6 different substitutions in 9 patients (9/59 GCs = 15.2%): 4 of the missense type (ID 10, ID 11, ID 12, ID 15) in 4 distinct patients (6.8%) and 2 of non-missense type (ID 2 and ID 17) in 5 distinct GCs (3.4%).

The ID 10 (p.G274S) is a novel missense mutation that we found in an old male with a GC mixed histotype. This variant was not detected in 187 free-cancer individuals (108BDs+59FDRs+20AMAGs) thus excluding a polymorphism. A pathogenic effect of ID 10 mutation was not supported after functional (aggregation and invasion) *in vitro* assays as we recently reported [26], nonetheless data from *in silico* characterization of the mutation and a reduction in β-catenin expression found in the tumor tissue cannot completely exclude the significance of this mutation in GC development. Thus, at today ID 10 remains a novel *CDH1* mutation with a pathogenesis of an undetermined significance.

The ID 11 (p.A298T) substitution in exon 7 of *CDH1* has already been described in a 36-year-old young Caucasian male in a HDGC family [27]. In our series, this variant was detected only in 1 male (S47) of 74-year-old with a mixed histotype. The potential pathogenic effect of this mutation has been confirmed through *in vitro* functional studies in different laboratories [27], [37], [38]. Here first modelling results (Figure 2A–B) by analyzing 3D protein-ligand binding interactions, strongly support the potential for altered protein function and lead to the possible molecular mechanism that sustain this process. The potential altered protein function was supported also from SIFT analysis (Table 4) with a good score. Moreover, a recent study, using the *in silico* protein design FoldX algorithmic approach [39], reasserts the pathogenic role of the ID 11 (p.A298T) substitution, based on a calculation of native-state stability changes (ΔΔG>0.08 kcal/mol) [40]. Authors characterized patients harbouring this missense mutation as having a younger age at diagnosis and a diffuse histotype. Our case highlighted that ID 11 can also be detected in an old patient with mixed GC.

The ID 12 (p.T470I) was found in a 57-year-old male (S39) with a diagnosis of GC. This change was first described in a family of Maori ethnicity with EOGC, but the subject showing this mutation was not affected by GC at the time of study [28]. Here, we found that the p.T470I AA change is tolerated by SIFT and also by modelling analysis. Unfortunately, the tumor bioptic tissue specimen was insufficient to perform E-cadherin IHC staining.

The ID 15 substitution (p.A592T) was detected in each clinical group tested, suggesting a probable polymorphic diffusion. Nonetheless, this variant has been previously reported associated with thyroid tumors and lobular breast cancers [41]–[43]. Our structural analysis and *in vitro* [35] and *in silico* studies [38], [40] do not support a pathogenic role for this variant in GC.

As recommended by recent clinical management guidelines [44], endoscopy surveillance should be performed annually in those individuals with mutations of undetermined significance (eg, missense). In our opinion, subjects harbouring ID 15 and also ID 10, must be followed for up to 10 years before excluding a role although weak for this alteration in the pathogenesis of GC.

In the ID 2 we identified a C-to-G change before the start codon (−71C>G, *CDH1* 5′UTR region), that represented the most common variant associated with GC in our series, occurring in three out of 59 GC patients (5.1%). This variant was also reported in a Finnish study [45] in 1 of 13 (7.7%) GC patients and in 2 of 51 controls (3.9%), and also in two EOGC patients of Northern American origin (3.4%) [46]. Overall data from these studies suggest that ID 2 is a quite common mutation but authors did not report data about ID 2 variant in relation to the E-cadherin expression status. ID 2 was found in our series in one intestinal, one mixed, and one diffuse GC histotypes. All these patients had over 50 years at diagnosis and were negative for HP infection. None of control subjects (n = 111) tested without GC, showed this mutation (Table 3). An *in situ* evaluation or a correlation between ID 2 and E-cadherin expression was unable to be performed due to a lack of tumor material. The potential pathogenic effect of this promoter variant on E-cadherin expression level deserves further studies.

Intronic ID 17 variant (IVS12 c.1937-13T>C) was found in 2 females with GC (2/59 GCs = 3.4%) both positive for HP infection, and it was found also in 1 BD (1/52 = 1.9%, Table 3). The same alteration was previously reported in lobular breast cancer with high frequency (12/53 = 23%) [47], in HDGC families (2/27 = 7.4%) [48] and in EOGC patients (7/79 = 8.9%) [46] but also in a relative control population [46]. Of note, we demonstrate for the first time that this substitution leads to an aberrant *CDH1* transcript harbouring a deletion of the *CDH1* exon 11. Exon 11, together with partial sequences of the flanking exons, codifies for the EC4 domain of the mature protein [25]; ID 17 is an out-of-frame deletion and leads to the formation of a premature stop codon at position 384 of the EC4 promoter. Consequently, the translated protein from *CDH1* ID 17 strand could lack the transmembrane domain and the cytoplasmic tail that is involved in β-catenin binding. Both S10 and S46 GC patients, having the ID 17 mutation, showed a reduction in the expression of E-cadherin and β-catenin by IHC analyses (Figure 5); the GC S10 patient, with a signet ring cell carcinoma, was diagnosed at the age of 61 years, and the GC S46 patient, with a diffuse adenocarcinoma, was diagnosed at the age of 58 years. Moreover, evaluation of E-cadherin expression from the EBV immortalized B-lymphocytes showed a strong reduction (60%) in GC S10 harbouring ID 17 mutation as compared with the BD control (S189) without *CDH1* alterations, but also as compared to a single blood donor (S190) carrying the same ID 17 variant. Since all subjects carrying the ID 17 mutation are heterozygous for the *CDH1* gene, our data indicated that the S190 individual, but not tumor cells of S10 and S46 patients, may exploit some compensatory mechanism that counteracts the E-cadherin down-regulation. In tumors, E-cadherin under-expression is linked to enhanced β-catenin transcriptional activity, a main effector of the Wnt pathway [49]. The expression of a large number of genes related to tumor progression, including those for cyclin D1, c-*myc*, vascular endothelial growth factor, and survivin is controlled via the Wnt/β-catenin pathway [50]. E-cadherin binding to β-catenin prevents its translocation to the nucleus; accordingly, a reduction of E-cadherin expression may favour GC pathogenesis through an increased nuclear β-catenin accumulation. Since patients S10 and S46 with ID17 variant are both women showing a helicobacter pylori (HP) infection, we assume that ID 17 might be associated with a sex-specific prognostic factor (it is well known that the incidence of GC is higher for men than for women) and/or an HP infection. A deletion of exon 11 in the *CDH1* gene was also described in a HDGC patient, but in this last case aberrant splicing was associated to a different intronic mutation (IVS11 c.1711+5G>A) [27]. Intriguingly, an alternatively spliced, non-functional E-cadherin transcript that lacks exon 11 of the gene had also been reported in some head and neck cancer cells [51] and chronic lymphocytic leukaemia cases (CLL) and although at a lower level compared to CLL, also in normal B cells [52]. In these cases no genetic alterations in exon 11 or in its flanking intronic regions were observed; the non-functional transcript has a premature termination codon and is degraded by the nonsense-mediated RNA degradation [52]. Splicing factors, binding in the region of exon 11 of *CDH1*, could have altered expression levels or states of activation in CLL cells compared with normal B-cells as recently demonstrated [53].

In the FDR group of subjects, with the exclusion of polymorphic and silent *CDH1* mutations, we observed 3 substitutions (ID 1, ID3 and ID 9) which were not found in GC.

The ID 1 (5′near gene-176C>T) variant was detected in a 32-year-old female with an unknown HP infection status. This variant was already submitted in popular databases, but its significance is unknown.

The ID 3 (5′UTR-54G>C) variant was found in a 72-year-old man positive for HP infection. Of interest, this mutation was already detected in a healthy 41-year-old Japanese subject, with no clinically detectable tumor at the time of the enrolment, and described as a rare variant able to decrease the transcriptional activity of *CDH1* [54]. We hypothesize that this mutation by introducing a CpG island in the *CDH1* promoter region increases the probability of *CDH1* hypermethylation, a well known event favouring the transcriptional inactivation, an early event in HP gastritis [55] and a key risk factor associated to GC development.

The ID 9 (intronic IVS4 c.532-18C>T) was found in a 41-year-old male subject negative for HP infection. ID9 was first reported in two EOGC patients from England and Portugal, respectively [56], in two HDGC German patients and in 1 control subject enrolled in the same study [35]. Recently, a non pathogenic role for this variant was proposed [2]. We did not notice any influence on *CDH1* splicing.

AMAG patients have a 3-fold increased relative risk of developing GC and have been never investigated for *CDH1* germline mutations until now. In AMAG series we found only polymorphic variants with the exception of ID 5, a new intronic mutation close to exon 1 (IVS1 c.48+7C>T). ID 5 was found in a female of 51-year-old with hypergastrinemia. We did not find any truncations or frameshifts in the production of the protein associated to this mutation. Although our series is limited (n = 20), these data seems not to support a relevant role of *CDH1* genetic alterations associated with AMAG disease.

In conclusion, our results show that the well known pathogenic ID 11 mutation (p.A298T) can also be detected in sporadic GC patients without fulfilling the strict criteria for HDGC. Furthermore, we demonstrated a deleterious effect of ID 17 variant (IVS12 c.1937-13T>C) on *CDH1* splicing and a related decrease in E-cadherin expression and also for β-catenin. The same ID 17 mutation and splicing effect found in 1 blood donor, but with a limited effect on E-cadherin mRNA level, is intriguing and deserves further studies. Considering the correlation among specific *CDH1* germline alterations and the tumor histotype, we found that 8.3% (1 of 12 GCs) of mixed (ID11) and 7.7% (2 of 26 GCs) of diffuse (ID17) subtypes, carried a potential pathogenic mutation.

Finally, in a FDR individual at risk for GC, we found the ID 3 variant (5′UTR-54G>C) with a potential effect of increasing the hypermethylation status of *CDH1*, a well known risk event associated with GC development and progression.

North East of Italy presents high GC incidence and mortality rates although lower respect to central regions, like Tuscany and Marche [57]. Our findings show prevalence in missense *CDH1* substitutions versus non-missense alterations, as reported in a recent metanalysis for middle-high GC risk areas like the Central Italy [58]. However, we can not excluded that the middle-high GC prevalence herein found might be slightly more likely than in the rest of Friuli geographic region since study was conducted in a Cancer Institute.

Moreover, variants found in subjects at risk for GC, particularly in FDRs and recently findings of novel mutations in sporadic GC patients in Chinese population [59], invite to screen for *CDH1* genetic alterations in addition to other risk factors, to define a high-risk group of patients that would benefit from an early GC diagnosis.

## Methods

### Patients and sample preparation

Fifty-nine patients at first GC diagnosis were consecutively recruited at the Gastroenterology Unit of Centro di Riferimento Oncologico (CRO), National Cancer Institute. Histopathological diagnoses were based on the WHO Classification [60] and Lauren's classification [7]. Clinicopathological characteristics of patients are reported in Table 1. Concurrently, 20 consecutive AMAGs (S119–S139) and 59 FDR (S60–S118) individuals (parents, children, siblings, and offspring of a relative with a GC) were recruited from the same centre; the participants inclusion criteria were patients who attended gastroenterology unit for gastric symptoms and with exclusion of a GC after endoscopic and histological tissue evaluation (Table 2). A random sampling of 52 (S139–S190) healthy blood donors is used to be representative of the general population (BDs). 56 additional BD controls were genotyped for the novel variants. For each participant, a peripheral blood sample was collected in acid citrate dextrose (ACD) tubes, and genomic DNA extracted using the EZ1 DNA Blood kit and the BioRobot EZ1 Workstation (QIAGEN Inc., Valencia, CA, USA). Multiple biopsies were collected for preservation and immunohistochemical analysis. All subjects freely gave their written informed consent. Ethical guidelines for research involving human subjects were respected and this study was approved by the CRO institutional review board (CRO: Ricerca corrente. Project n.4 linea n.1).

### Germline *CDH1* mutation screening

PCR *CDH1* primer sequences for the amplification of all 16 coding exons were previously reported. PCR reactions were carried out in a volume of 10 µl containing 10 ng of genomic DNA template, 1 mM MgCl_2_, 1 mM dNTPs, 0.6 µM of each PCR primer, 5X Green Buffer and 0.25 U Go-Taq DNA Polymerase (Promega, Madison, WI, USA). Furthermore, 5% DMSO was added to PCR reactions for exon 1 and 2. Thirty cycles of 30 s at 94°C, 30 s at 60°C and 1 min at 72°C were performed in a programmable thermocycler (Eppendorf, Hamburg, Germany). A 2 µl aliquot of the PCR product was then purified using 0.5 µl of ExoSAP-IT kit (USB Corporation, Cleveland, OH, USA), and a 0.5 µl aliquot of this purified product was sequenced using the Big Dye Terminator kit (Applied Biosystems, Foster City, CA, USA) on an ABI PRISM capillary sequencer. Chromas and ClustalW software were used for multiple sequence alignment. Variants detected were confirmed using the genomic DNA sequence.

### Characterization of the impact on splicing for intronic variants by RT-PCR

To detect intronic splice variants, RNA was isolated from the peripheral blood mononuclear cells (PBMC) of patients with a *CDH1* germline alteration and that of control patients negative for the same variant using the EZ1 RNA Cell Mini Kit and the BioRobot EZ1 Workstation (QIAGEN Inc.). First-strand cDNA was synthesized from 0.8 µg total RNA with the High Capacity cDNA Reverse Transcription Kit (Applied Biosystems) according to the manufacturer's protocol. *CDH1* transcripts were amplified using gene-specific forward (FP) and reverse (RP) primers: i) *CDH1* exons 1–5 flanking the c.IVS1+7C>T and c.IVS4-18C>T sequence variants (FP: 5′-GGAAGTCAGTTCAGACTCCAGCC-3′ and RP: 5′-GTGGCAATGCGTTCTCTATCCAG-3′); ii) the *CDH1* exons 10–13 flanking the c.IVS12-13T>C variant (FP: 5′-ACCGTCACCGTGGATGTGCT-3′ and RP: 5′-GAATCCCCAGAATGGCAGGAA-3′). 5% DMSO was added to exons 1–5 PCRs. PCR product sizes were checked against a DNA ladder (Marker VIII, Roche Applied Science, Indianapolis, IN, USA) on a 4% agarose gel, and then sequenced [2].

### Immunohistochemistry

Formalin-fixed, paraffin-embedded tissue blocks (tumor and non-tumor) from patients carrying a *CDH1* germline mutation were cut into 5 µm-thick sections for H&E staining and immunostaining. Immunohistochemistry (IHC) was performed using the mouse monoclonal antibody against human E-cadherin (clone 36, Ventana Medical System, Tucson, AZ, USA), and β-catenin (clone 17C2 Novocastra, Newcastle upon Tyne, UK). H&E staining was performed according to standard protocols. Appropriate positive and negative control samples were included with each staining series.

### Structural modelling and *in silico* characterization of missense variants

Structural studies on the effect of the missense variants were performed using Pymol (http://pymol.sourceforge.net/ Accessed 16 January 2012) and WinCoot [61]. For the p.A298T in the EC2 domain, we used the scaffold of the crystal structure of the corresponding human wild-type E-cadherin protein (PDB code: 2O72) [25] For the two missense variants (p.T470I and p.A592T located in EC3 and EC4, respectively), we used the murine crystallized sequence (PDB code: 3Q2V) [62].

To test a prediction value of the phenotypic effect of the genetic mutations, an *in silico* analysis was performed using the SIFT (Sorting Intolerant From Tolerant, http://sift.jcvi.org/ Accessed 21 April 2011) algorithm [63] and the polymorphism phenotyping Polyphen-2 tool (http://genetics.bwh.harvard.edu/pph2/ Accessed September 1, 2011). E-cadherin protein sequence (GI: 31073) was used for alignment comparisons and only mutations with a score below 0.05 were considered to be intolerant for SIFT. The A8K1U7_HUMAN feature (UniProtKB/TrEMBL) was used with the Polyphen tool.

### Cell lines and relative quantitative real-time RT-PCR

Lymphoblastoid cell line (LCLs) from four subjects (GC S10, FDR S97, BD189 and BD190) were generated by *in vitro* immortalisation of B cells with the B.95.8 Epstein-Barr virus isolate [64]. Cell lines were cultured in RPMI-1640, containing 10% heat-inactivated FBS (Gibco-BRL, Gaithersburg, MD, USA), 2 mmol/l L-glutamine, 100 mg/ml streptomycin, and 100 IU/mL penicillin (Sigma-Aldrich, St. Louis, MO, USA) and maintained at 37°C in 5% CO_2_. LCLs were generated from the seeding of 2,5×10^6^ PBMC. About 8 million of cells were harvested for each sample after immortalization. Total RNA was isolated and used to synthesize cDNA, as described above. Relative quantitative real-time RT-PCR for E-cadherin expression was performed with 2X SYBR Green Master Mix (Applied Biosystems) using a 7500 Real Time PCR system (Applied Biosystems). Primers (set ii) and amplification were as described above. The Ex10-13 product determined the wild-type E-cadherin transcription level. Normalisation of RT-PCR products was determined using the Pfaffl method with β-actin (ACTB) (FP: 5′-GACCCAGATCATGTTTGAGA-3′; RP: 5′-GACTCCATGCCCAGGAAG-3′) as the endogenous control and BD S189 as the reference sample. All experiments were run in triplicate and the mean values were used to calculate E-cadherin mRNA expression.

### Statistical analysis

Results obtained in triplicate were expressed as the mean ± SD. Differences between groups were determined by unpaired *t*-tests. A *p*-value of less than 0.05 was considered significant.
